# How Active Are Women in the First Year of Motherhood? A Systematic Review of Device‐Measured Physical Activity

**DOI:** 10.1111/sms.70108

**Published:** 2025-07-31

**Authors:** Freja Hauberg Hallen, Sebastian Dyrup Skejø, Rasmus Oestergaard Nielsen, Solvej Videbæk Bueno

**Affiliations:** ^1^ Research Unit for General Practice Aarhus Denmark; ^2^ Department of Public Health Aarhus University Aarhus Denmark

**Keywords:** device‐measured physical activity, motherhood, MVPA, physical activity, postpartum

## Abstract

This systematic review summarizes previous findings of device‐measured moderate‐ and vigorous‐intensity physical activity (MVPA) in minutes per day from childbirth to 1 year postpartum. A systematic literature search was conducted in PubMed and Embase. Eligible studies reported device‐measured MVPA at any time within the first 12 months postpartum in healthy parous women. Out of the 1437 studies identified, 15 studies were included, with study populations ranging from 20 to 532 women. MVPA was measured using various physical activity (PA) trackers, with ActiGraph devices being the most common (*n* = 10). Definitions of MVPA varied, utilizing counts per minute (cpm), Euclidean Norm Minus One (ENMO), metabolic equivalent tasks (METs), or proprietary algorithms. MVPA estimates ranged from 2.14 to 87.3 min/day. Most studies revealed an estimate of MVPA ≤ 27.6 min/day (*n* = 10), while four studies reported ≥ 55.5 min/day. MVPA estimates varied widely, with most studies reporting estimates below the World Health Organization recommendation of a minimum of 150 min MPA per week. Differences in study characteristics, tracker placement, data processing, and the cut‐offs used to define MVPA likely contributed to variability, highlighting the need for standardized methodologies to improve comparability. Expanding our knowledge of postpartum PA can support new mothers in meeting health‐enhancing PA recommendations.

## 1 Introduction

Physical activity (PA) is widely recognized as a factor that contributes to maintaining overall health. Research suggests that PA supports both physical and mental well‐being and is associated with beneficial outcomes in relation to chronic diseases, such as cardiovascular conditions, diabetes, and some cancers [1, 2, 3]. Additionally, PA is linked to improved mental health, including reduced symptoms of depression and anxiety [2, 4]. Given the evidence of suggested health benefits, the World Health Organization (WHO) recommends that adults engage in 150–300 min per week (min/week) of moderate‐intensity physical activity (MPA), 75–150 min/week of vigorous‐intensity physical activity (VPA), or an equivalent combination of moderate‐ and vigorous‐intensity physical activity (MVPA). In 2020, WHO issued specific recommendations for pregnant and postpartum women, advising at least 150 min of MPA per week, incorporating both aerobic and muscle‐strengthening activities. This marked the first instance in which WHO provided tailored guidelines for postpartum women [5].

Postpartum women engage in lower levels of PA than recommended [6, 7]. A recent Danish population‐based study assessed self‐reported PA and found that mothers had a 24% higher risk of non‐adherence to WHO PA guidelines compared with their nulliparous peers [8]. In line with these findings, previous literature based on self‐reported questionnaires has also reported a decline in PA from pre‐pregnancy to the postpartum period [9, 10]. This is concerning, as PA during the postpartum period has been linked to numerous health benefits, including enhanced well‐being [11, 12], reduced postpartum weight retention [13] and lower risk of postpartum depression [14, 15, 16]. To support postpartum women in achieving recommended PA levels, it is essential to understand PA behaviors during this period, which can be assessed using either self‐reported or device‐measured PA.

Device‐measured PA is increasingly used in research to enhance the assessment of PA levels [17]. PA trackers are wearable devices, usually placed at the hip or wrist, most of them using accelerometry to measure movement across multiple axes [18, 19], while others combine accelerometry with additional sensors or algorithms to estimate PA intensity and duration [20]. Compared to self‐reported measures, PA trackers offer the advantage of eliminating recall bias, as they do not rely on participants' ability to recall and report their activity levels [21]. However, device‐measured PA also has limitations, such as limited contextual detail and methodological variability [17]. Despite the advantages of device‐measured PA, research examining postpartum PA using these methods remains limited [22, 23].

To address this gap, the aim of the present systematic review was to compile the evidence from original articles describing device‐measured MVPA in postpartum women, reported in minutes per day, from childbirth to 1 year postpartum.

## 2 Methods

### 2.1 Search Strategy

The present review was conducted according to the Preferred Reporting Items for Systematic Reviews and Meta‐Analyses (PRISMA) guidelines [24].

A comprehensive literature search was conducted on March 16, 2023, and updated on November 6, 2024, using the following electronic databases: Embase and PubMed. The search strings were developed in collaboration with a certified librarian from the Royal Danish Library and included a combination of free‐text terms and Medical Subject Headings (MeSH) terms related to physical activity, the postpartum period, and health. The full search protocols from each database are listed in Data S1 and S2. The completed PRISMA checklist is available in Data S3.

### 2.2 Eligibility Criteria

Studies were eligible if they reported device‐measured MVPA or provided separate estimates for MPA and VPA at any time within 12 months postpartum in healthy parous women following a singleton gestation, regardless of age, education, race, culture, and ethnicity. Only primary literature published in English or Danish after the year 2000 was included. All original study designs were accepted.

Eligible studies were excluded if they met one or more of the following exclusion criteria: (1) did not measure PA with PA trackers, (2) did not report PA data measured within 12 months postpartum, (3) did not report MVPA in min/day or min/week, (4) provided information about PA only for an intervention group, (5) cohorts were selected based on psychiatric or medical conditions, (6) used the same data as an included study.

### 2.3 Study Selection

One author (FHH) assessed the eligibility using the browser‐based screening tool Covidence (Veritas Health Innovation, Melbourne, Australia. Available at: www.covidence.org). Screening was conducted in two stages. First, titles and abstracts were screened, followed by a full‐text assessment of eligible studies. In cases of uncertainty, the author consulted co‐authors to discuss these uncertainties. Additional citation searching was performed by reviewing reference lists of included studies and searching the database Scopus for further exploration.

### 2.4 Data Collection

FHH collected the following data from each included study: author and year of publication; study country; study design; size of study population and PA measure time points; and characteristics of participants.

In this review, the term PA trackers refer to the devices used to objectively measure PA. Detailed data on PA tracker types, definitions of MPA or MVPA, and wear‐time were extracted by FHH to provide an overview of the methods used to assess PA intensity.

Estimates of MVPA or separate estimates for MPA and VPA were extracted from the included studies and summarized, using the format reported in each study (e.g., mean (SD), mean (SE), mean (95% CI), or median (IQR)). For one study, the statistical measure used was not specified, and the data are presented as described in the original publication [23]. When studies reported MVPA at multiple time points, all eligible estimates within the first year postpartum were included. Some studies presented the same estimates of MVPA using multiple cut points. For this review, only one estimate per dataset was included, prioritizing the cut points most comparable across all included studies using cpm to define MPA or MVPA. MPA or MVPA was visually presented (Figure 2). A complete dataset summarizing all extracted data from included studies is provided in Data S4.

No formal quality assessment or risk of bias tool was applied, given the descriptive aim and heterogeneity in study designs.

### 2.5 Statistical Analysis

For studies that included both an intervention group and a control group, only data about the control group was used to reflect PA patterns in a population unexposed to an intervention. In studies that divided the population into subgroups (e.g., by ethnicity or sleep patterns), weighted means (weighted by 1/SE^2^) were calculated to provide estimates across subgroups. For studies that provided mean (SD) for time of measurement postpartum, a 95% prediction interval (PI) was calculated. The weighted means and 95% PI were calculated using a custom Microsoft Excel spreadsheet. MVPA estimates originally reported as minutes per week (min/week) were converted to minutes per day (min/day) by dividing by seven to estimate the duration of PA per day.

## 3 Results

### 3.1 Study Selection

**FIGURE 1:**
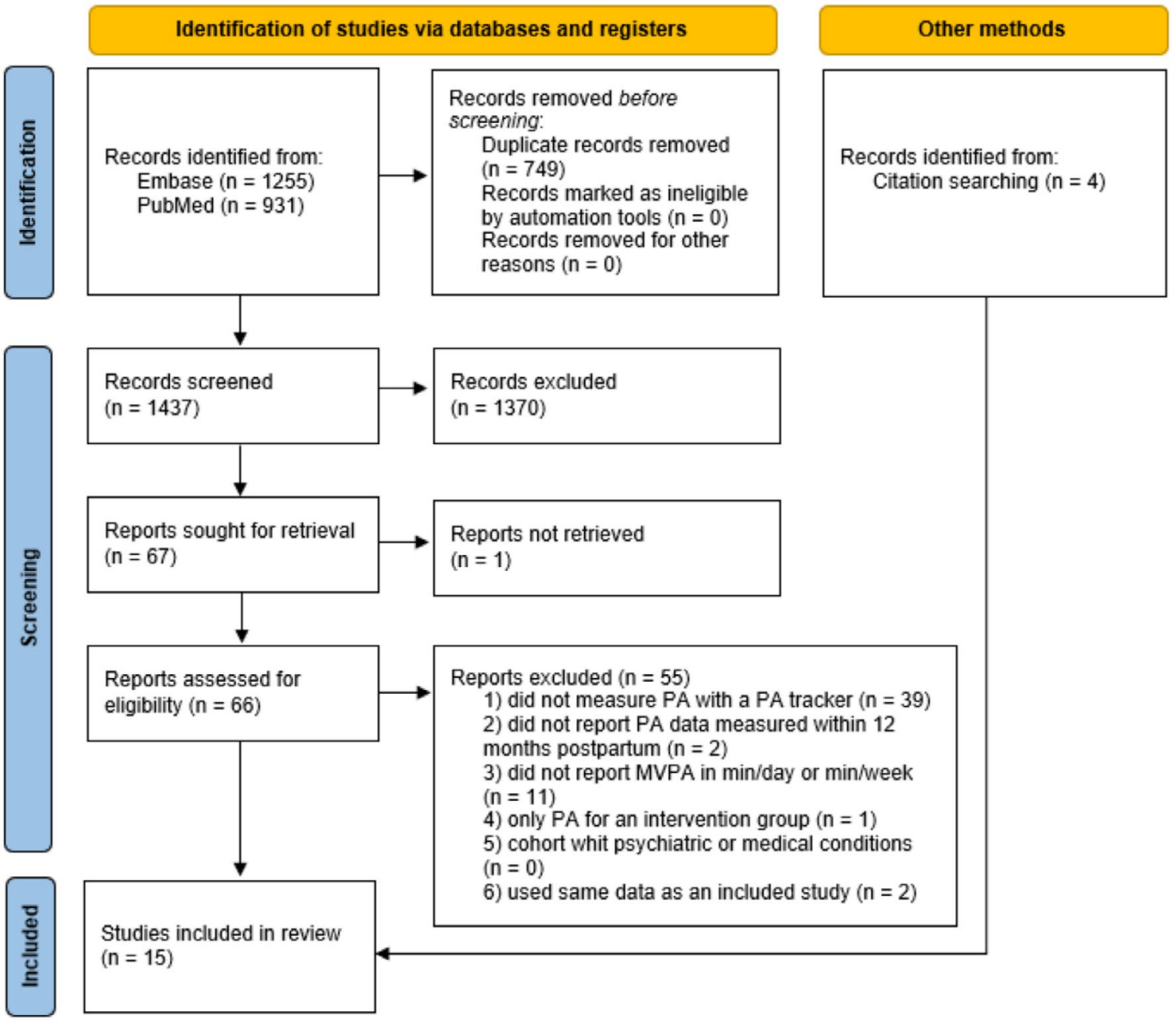
Flowchart of the study selection process. MVPA, Moderate‐ and vigorous‐intensity physical activity; min/day: Minutes per day; min/week: Minutes per week; N, number; PA, physical activity.

A flowchart of the study selection is presented in Figure 1. The database search identified 2186 records (1255 from Embase and 931 from PubMed). After excluding duplicates, the titles and abstracts of 1437 records were screened. Of these, 66 reports were selected for full‐text screening to assess eligibility. Fifty‐five reports were excluded, leaving 11 studies to be included [23, 25, 26, 27, 28, 29, 30, 31, 32, 33, 34]. From citation searching, four additional studies were included [22, 35, 36, 37]. In total, 15 studies were included in the present review.

Most reports were excluded from the full text screening due to lack of device‐measured PA. Eleven reports that used device‐measured PA but did not report MVPA in min/day or min/week were also excluded. Two of these 11 reports used pedometers [38, 39]. To avoid duplication bias, 2 reports were excluded because they used the same data as included studies [40, 41].

### 3.2 Study Characteristics

Information on study and population characteristics for included studies are found in Table 1. All included studies were published between 2012 and 2024. Eleven studies were conducted in the USA, while the remaining four were from Australia, Japan, Finland, and Norway, respectively. Eight studies were cohorts, four were randomized controlled trials, and three were cross‐sectional studies. Eight studies did not have PA as their primary outcome, but all reported MVPA data. Two studies presented MPA and VPA separately.

The sample size ranged from 20 to 532 women, with study populations varying by ethnicity, socioeconomic status, and body mass index (BMI). The mean age for women in the individual studies ranged from late twenties to early thirties. Five of the study populations had a high prevalence of overweight or obese individuals, and several studies focused on low‐income or ethnic minority groups.

**TABLE 1:** Description of included studies.

Author (year)	Study country	Study design	Size of study population and PA measurement time point	Characteristics of participants
Bennetter et al. (2023) [35]	Norway	Cohort study	*N* = 169 minority ethnic women had PA measured between 2.07 and 4.34 months postpartum^b^	Mean age 29.5 (SD = 4.9), mean BMI 25.3 (SD = 5.0), 95% were married/cohabitant, 41% South Asia ethnicity, 41% had high school/secondary education
Evenson et al. (2012) [25]	USA	Cohort study	*N* = 181 women had PA measured at 3 months postpartum	55% were ≥ 30 years, 85% were married, 73% were non‐Hispanic white, 30% were overweight
			*N* = 204 women had PA measured at 12 months postpartum	71% were ≥ 30 years, 90% were married, 82% were non‐Hispanic white, 45% were overweight
Evenson et al. (2013) [26]	USA	Cohort study	*N* = 132 obese or overweight women had PA measured between 3.45 and 6.21 months postpartum.	50% were between 30 and35 years, 81.1% were non‐Hispanic white, 93.9% were married, 58.3% were obese
Hesketh et al. (2018) [36]	USA	Cohort study	*N* = 55 low‐income, predominately black women, had PA measured at 3 months postpartum	71% were between 21 and 30 years, 4% were Hispanic/Latina, 77% were Black/African American, 25% were married, 39% were obese pre‐pregnancy, 64% had an annual household income ≥ $20 000
			*N* = 55 low‐income, predominately black women, had PA measured at 9 months postpartum	
Keller et al. (2014) [27]	USA	Randomized Controlled Trial	*N* = 54 sedentary^a^ obese or overweight Latinas, had PA measured between 1.38 and 6 months postpartum	Mean age 28.3 (SD = 5.59) years, first pregnancy for 20.1%, 121 of the participants were born in Mexico but lived in USA. 69.3% had a household income ≥ $20 000
			*N* = 54 sedentary^a^ obese or overweight Latinas, had PA measured between 7.38 and 12 months postpartum	
Kernot et al. (2019) [28]	Australia	Randomized Controlled Trial	*N* = 40 controls had PA measured between 4.8 and 7.2 months postpartum	Mean age 30.7 (95% CI 29.2–32.2) years, 95% were married, 77.5% were not working/maternity leave
			*N* = 40 controls had PA measured between 6.3 and 8.7 months postpartum	
Kishman et al. (2023) [22]	USA	Longitudinal cohort study	*N* = 130 women had PA measured between 1.38 and 1.84 months postpartum	Characteristics of enrolled participants (*N* = 134); mean age 30 years, 63% were White, 39% were obese, 59% had graduated college or above, 88% were in a stable relationship, 50% of the participant had a household income > US$ 60 000, 73% had a vaginal delivery
			*N* = 109 women had PA measured at 4 months postpartum	
			*N* = 99 women had PA measured at 6 months postpartum	
			*N* = 75 women had PA measured at 9 months postpartum	
			*N* = 77 had PA measured at 12 months postpartum	
Kracht et al. (2024) [29]	USA	Secondary analysis of a control group from a Randomized Controlled Trial	*N* = 150 women had PA measured ~1 year postpartum	Mean age 31.3 (SD = 3.5) years, 53.5% were Black or Hispanic, 45.1% were overweight during early pregnancy
Lewis et al. (2024) [30]	USA	Randomized controlled study	*N* = 55 low‐income women had PA measured between 1.84 and 2.76 months postpartum	Mean age 31.44 (SD = 5.31), 58.2% were Caucasian, 40% were single (never married), 73% had a household income < US$40000
Marshall et al. (2020) [31]	USA	Cohort study	*N* = 23 women had PA measured 2.76 months postpartum	Mean age 31 (SD = 3.70) years, weight 68.83 (SD = 7.81) kg, BMI 24.58 (2.81) kg/m^2^
Martin et al. (2017) [32]	USA	Cross‐sectional study	*N* = 282 obese or overweight who all identified as Hispanic had PA measured 5.4 (SD = 3)^c^ months postpartum	Mean age 28 years, 60% were born in Mexico, 34.8% were obese
Saarikko et al. (2020) [33]	Finland	Cohort study	*N* = 20 women had PA measured the first month following birth	Mean age 26 years (SD = 5.0). 45% had a secondary education, 15% were single, 65% were working, pre‐pregnancy BMI median 24.4 kg/m^2^
Shaw et al. (2023) [37]	USA	Prospective cohort study	*N* = 532 women had PA measured between 5 and 8 months postpartum	Mean age 29.9 (SD = 4.9) years, 91.7% were White, 84.8% non‐Hispanic ethnicity, 67.4% had a BMI < 25
Tomioka et al. (2022) [23]	Japan	Cross‐sectional study	*N* = 99 women had PA measured at 2 months postpartum	Mean age 32.14 (SD = 3.82) years, none worked at the time of measure. Mean BMI 21.04 (SD = 2.03) kg/m^2^
Wolpern et al. (2021) [34]	USA	Cross‐sectional study	*N* = 500 primiparous women had PA measured 0.46–0.69 months postpartum	Mean age 28.3 (SD = 5.1) years, pre‐pregnancy BMI median 23.5 kg/m^2^, 80.6% white, 21.1% Hispanic or Latina
			*N* = 473 primiparous women had PA measured 1.15–1.38 months postpartum	

*Note:* PA was measured within the specified postpartum periods. Participants were instructed to wear the PA tracker for 1 week within the given timeframe. Except in Bennetter et al. (2023) [35]; instructed wear‐time 4–7 days, Martin et al. (2017) [32]; instructed wear‐time 5–7 days, Lewis et al. (2024) [30]; instructed wear‐time until 3 months postpartum, Saarikko et al. (2020) [33]; instructed wear‐time 1 month, Tomioka et al. (2022) [23]; instructed wear‐time 2 days. Measure of PA after 1 year postpartum is not mentioned.

Abbreviations: BMI, body mass index; MVPA, moderate‐ and vigorous‐ intensity physical activity; N, number of participants; PA, physical activity; SD, standard deviation.

^a^ Sedentary definition used in these studies: < 2.5 h of moderate‐intensity PA per week.

^b^ 95% prediction interval from mean (SD) reported in study.

^c^ Weighted mean across study groups.

### 3.3 Description of PA Trackers

Table 2 summarizes PA tracker data and wear‐time across the included studies. Seven different PA trackers were used, with ActiGraph models being the most common. Trackers were primarily placed on the hip (*n* = 8) or wrist (*n* = 5), with one study using both placements and another using the upper arm. MVPA definitions varied, with seven studies using counts per minute (cpm), while others applied alternative cut‐offs, including Euclidean Norm Minus One (ENMO), heart rate thresholds, steps per minute, metabolic equivalent tasks (METs) or proprietary algorithms. Wear‐time also differed, with most studies (*n* = 10) instructing participants to wear trackers for 7 days, while in others, instructed wear‐time ranged from 2 days to 3 months postpartum. Criteria for valid wear‐time typically required ≥ 3 days with ≥ 10 h per day (h/day), though variations existed. Reported mean valid wear‐time ranged from 11.5 to 23.5 h/day or 4 to 15 days. Two studies did not specify valid data criteria.

**TABLE 2:** Description of physical activity tracker types, definitions of physical activity, and data regarding wear‐time.

Author (year)	PA tracker type	Placing	Definition of MPA or MVPA	Inclusion criteria for data	Mean wear‐time for valid data	Instructed wear‐time
Bennetter et al. (2023) [35]	SenseWear Pro3	Upper arm	MVPA ≥ 3 METs	≥ 19.2 h/day for ≥ 2 days	Ranged from 23.3 to 23.5 (SD 0.5–0.7) h/day	4–7 days
Evenson et al. (2012) [25]	ActiGraph model #7164	Hip	MPA 2020–5998 cpm; VPA > 5999 cpm^c^	Used all data	3 months: 12.2 h/day^a^	7 days
					12 months: 12.8 h/day^a^	
Evenson et al. (2013) [26]	The Actical	Hip	MVPA > 1535 cpm^d^	≥ 6 h/day for ≥ 4 days (including 1 weekend day and two weekdays)	13.3 (IQR 11.8–14.4) h/day^a^	7 days
Hesketh et al. (2018) [36]	ActiGraph GT3X+	Hips + wrist	MVPA > 2020 cpm^c^	≥ 3 days of measurement Wrist: ≥ 10 h/day Hip: ≥ 8 h/day	hip: 13.1 (SD 1.8) hours across 5.7 (SD 1.7) days	7 days, 24 h/day for wrist and the hip only during waking hours
					wrist: 16.2 (SD 1.9) hours across 7.4 (SD 1.5) days	
Keller et al. (2014) [27]	ActiGraph GT1M	Hip	MPA: 1952–5725 cpm; VPA ≥ 5725 cpm^e^	≥ 3 days with ≥ 10 h/day	At baseline: 14.2 h/day	7 days at baseline and then 7 days 6 month later
Kernot et al. (2019) [28]	ActiGraph GT3X+	Hip	MVPA: 2020–5998 cpm^c^	≥ 10 h/day for ≥ 4 days (including 1 weekend day)	No data	24 h/day for 7 days
Kishman et al. (2023) [22]	ActiGraph GT3X+	Hip	MPA 2020–5998 cpm; VPA > 5999 cpm^c^	≥ 10 h/day for ≥ 3 days	6 days (range 3–10 days) and 16.1 (SD 3.2) h/day	7 days at 5 different time points postpartum
Kracht et al. (2024) [29]	ActiGraph GT3X+	Wrist	MVPA ≥ 100 mg	≥ 20 h/day with > 10 h of awake wear on ≥ 1 day	4 (SD 1.5) days	7 days
Lewis et al. (2024) [30]	Fitbit Flex	Wrist	Proprietary algorithms from Fitbit	≥ 10 h/day	No data	Entire trial (during pregnancy and until 3 months postpartum)
Marshall et al. (2020) [31]	ActiGraph GT3X+	Hip	MVPA ≥ 100 steps/min	≥ 3 days of wear with ≥ 8 h per day	11.5 (SD 1.4) h/day	7 consecutive days during waking hours
Martin et al. (2017) [32]	ActiGraph GT3X+	Hip	MPA: 2690–6166 cpm^f^	≥ 10 h/day	11.6 (SD 2.8) h/day^b^	5–7 days
Saarikko et al. (2020) [33]	Garmin Vivosmart	Wrist	MPA is defined as 70%–80% of maximum heart rate	10 h/day on ≥ 3 weekdays and 8 h/day on at least 1 weekend day	15 (range 0–25) days^a^	24 h/day during the month following birth
Shaw et al. (2023) [37]	ActiGraph GT9X	Wrist	MVPA ≥ 100.6 mg	≥ 10 h/day for ≥ 4 weekdays including ≥ 8 h/day for ≥ 1 weekend day	22.8 (SD 2.4) h/day across 7.1 (SD 1.5) days	7 days
Tomioka et al. (2022) [23]	Active Style Pro HJA‐750C	Hip	MVPA ≥ 3 METs	≥ 8 h/day for 2 days	12.55 h	24 h for 2 days
Wolpern et al. (2021) [34]	ActiGraph GT9X	Wrist	MVPA ≥ 100.6 mg	10 h/day on ≥ 3 weekdays and 8 h/day on at least 1 weekend day	0.5–0.75 months: 7.6 (SD 1.4) days and 23.3 (SD 1.6) h/day	24 h/day for 7 consecutive days
					1.25–1.5 months: 7.1 (SD 1.3) days and 23.0 (SD 2.2) h/day	

Abbreviations: Cpm, counts per minute; h/day, hour per day; IQR, inter quartile range; MET, metabolic equivalent of task; mg, milligravity; min/day, minutes per day; MPA, moderate intensity physical activity; MVPA, moderate‐ and vigorous‐ intensity physical activity; PA, physical activity; SD, standard deviation; steps/min, steps per minute; VPA, vigorous intensity physical activity.

^a^ Median value.

^b^ Weighted mean (SD).

^c^ Cut‐points defined by Troiano et al. [42]; MPA 2020–5998 cpm; VPA > 5999 cpm; MVPA > 2020 cpm.

^d^ Cut‐points defined by Colley et al. [43]; MVPA ≥ 1535 cpm.

^e^ Cut‐points defined by Freedson et al. [44]; MPA 1952–5724 cpm; VPA ≥ 5725 cpm.

^f^ Cut‐off points defined by Sasaki et al. [45]; MVPA ≥ 2690 cpm.

### 3.4 Study Outcome Measures

**FIGURE 2:**
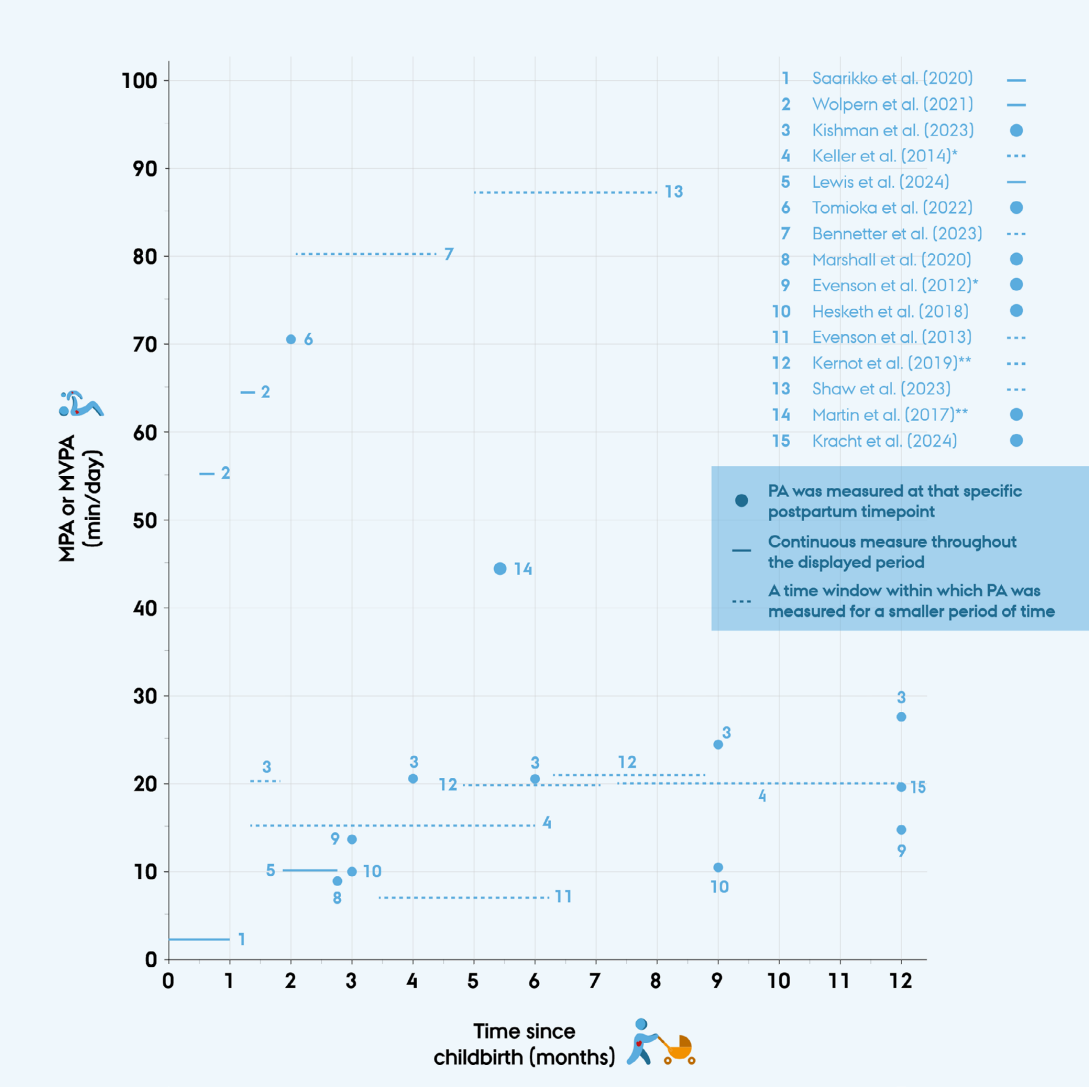
Mean or median daily moderate‐to‐vigorous physical activity (MVPA) from included studies, measured in minutes per day (min/day) using physical activity (PA) trackers and plotted by time since childbirth (in months). Each data point represents a reported MVPA or moderate physical activity (MPA) estimate. Numbers refer to study authors (see legend). Repeated numbers indicate that the same study reported MVPA estimates from multiple timepoints. *MPA and vigorous physical activity (VPA) were reported separately; only MPA is shown here (VPA < 1 min/day). **MVPA estimates are weighted means across study groups.

Estimates of MPA, VPA, or MVPA from childbirth to 12 months postpartum are displayed in Table 3 and Figure 2. A total of 24 MPA or MVPA estimates were reported across the 15 included studies, as some studies reported MVPA at multiple postpartum time points.

PA was measured within the specified postpartum periods listed for each study, but it is important to note that most participants were instructed to wear the trackers for only one week within the given timeframe. Only two studies [30, 33] instructed participants to wear the trackers continuously throughout the specified measurement period.

**TABLE 3:** Estimates of device‐measured physical activity in the included studies.

Time of measured PA after childbirth	Author (year)	Mean or median	PA estimates
0–1 months	Saarikko et al. (2020) [33]	Median (range)	MVPA: 2.14 (0–26.86) min/day^c^
0.46–0.69 months	Wolpern et al. (2021) [34]	Median (IQR)	MVPA: 55.5 (40.4–74.3) min/day
1.15–1.38 months	Wolpern et al. (2021) [34]	Median (IQR)	MVPA: 64.5 (47–84.6) min/day
1.38–1.84 months	Kishman et al. (2023) [22]	Mean (SE)	MVPA: 21.5 (3.1) min/day
1.38–6 months	Keller et al. (2014) [27]	Mean (SD)	MPA: 15.33 (12.28) min/day VPA: 0.16 (0.64) min/day
1.84–2.76 months	Lewis et al. (2024) [30]	Mean	MVPA: 10.8 min/day^d^
2 months	Tomioka et al. (2022) [23]	Not specified	MVPA: 71 min/day
2.07–4.34 months^b^	Bennetter et al. (2023) [35]	Mean (SD)	MVPA: 80.7 (63.9) min/day
2.76 months	Marshall et al. (2020) [31]	Mean (SD)	MVPA: 8.4 (8.7) min/day
3 months	Evenson et al. (2012) [25]	Median (IQR)	MPA: 13 (9–21) min/day VPA: 1 (0–0) min/day
3 months	Hesketh et al. (2018) [36]	Mean (SD)	MVPA: 10.0 (8.2) min/day
3.45–6.21 months	Evenson et al. (2013) [26]	Median (IQR)	MVPA: 6.9 (2.3–11.3) min/day
4 months	Kishman et al. (2023) [22]	Mean (SE)	MVPA: 22.1 (3.1) min/day
4.8–7.2 months	Kernot et al. (2019) [28]	Mean^a^	MVPA: 19.8 min/day^e^
5–8 months	Shaw et al. (2023) [37]	Mean (SD)	MVPA: 87.3 (35.5) min/day
5.4 months^a^	Martin et al. (2017) [32]	Mean (SD)^a^	MVPA: 44.7 (26.9) min/day
6 months	Kishman et al. (2023) [22]	Mean (SE)	MVPA: 22.2 (3.2) min/day
6.3–8.7 months	Kernot et al. (2019) [28]	Mean^a^	MVPA: 21.4 min/day^f^
7.38–12 months	Keller et al. (2014) [27]	Mean (SD)	MPA: 20.62 (16.87) min/day VPA: 0.66 (2.38) min/day
9 months	Hesketh et al. (2018) [36]	Mean (SD)	MVPA: 10.8 (8.7) min/day
9 months	Kishman et al. (2023) [22]	Mean (SD)	MVPA: 24.1 (3.3) min/day
12 months	Evenson et al. (2012) [25]	Median (IQR)	MPA: 17 (10–25) min/day VPA: 0 (0–1) min/day
12 months	Kishman et al. (2023) [22]	Mean (SE)	MVPA: 27.6 (3.2) min/day
12 months	Kracht et al. (2024) [29]	Mean (SD)	MVPA: 19.7 (0.7) min/day

*Note:* PA was measured within the specified postpartum periods. Participants were instructed to wear the PA tracker for 1 week within the given timeframe. Except in Bennetter et al. (2023) [35]; instructed wear‐time 4–7 days, Martin et al. (2017) [32]; instructed wear‐time 5–7 days, Lewis et al. (2024) [30]; instructed wear‐time until 3 months postpartum, Saarikko et al. (2020) [33]; instructed wear‐time 1 month, Tomioka et al. (2022) [23]; instructed wear‐time 2 days. Measure of PA after 1 year postpartum is not mentioned.

Abbreviations: CI, confidence interval; IQR, inter quartile range; min/day, minutes per day; min/week, minutes per week; MPA, moderate physical activity; MVPA, moderate‐vigorous physical activity; PA, physical activity; SD, standard deviation.

^a^ Weighted mean across study groups.

^b^ 95% prediction interval from mean (SD) reported in study.

^c^ Reported MVPA median (range): 15 (0–188) min/week.

^d^ Reported MVPA mean (SD): 75.33 (127.01) min/week.

^e^ Reported MVPA weighted mean (95% CI): 137 (102–173) min/week.

^f^ Reported MVPA weighted mean (95% CI): 150 (119–181) min/week.

Of the included studies, 10 reported MVPA as a mean estimate, four as a median estimate, and in one study [23], it was unclear whether the reported MVPA estimate represented a mean or median.

MVPA estimates varied widely across studies and ranged from 2.14 to 87.3 min/day, but with a mostly bimodal distribution. To reflect this pattern, MVPA estimates were grouped into three categories based on observed ranges: MVPA ≤ 27.6 min/day, MVPA between 27.6 and 55.5 min/day, and MVPA ≥ 55.5 min/day. This was done post hoc based on the bimodal distribution and visual trends observed in Figure 2. Notably, only one study [32] reported an MVPA estimate within the intermediate range (44.7 min/day), using a hip‐worn ActiGraph device and defining MVPA by cpm.

#### 3.4.1 Group With MVPA Estimates ≤ 27.6 Min/Day

This group included 18 MVPA estimates from the included studies, as some studies reported estimates from multiple postpartum timepoints. MVPA in this group ranged from 2.14 to 27.6 min/day, measured from childbirth to 12 months postpartum. The lowest estimate was reported from childbirth to 1 month postpartum [33], while the highest was measured at 12 months postpartum [22]. Fourteen estimates in this group came from studies that used hip‐worn ActiGraph devices with the same or similar cpm thresholds. Among the last four estimates in this group, two also used an ActiGraph device but had it placed at the wrist and did not define MVPA by cpm. The last two estimates came from studies using Fitbit and Garmin devices; these were placed at the wrist and defined MVPA differently than the other studies. Visual inspection of Figure 2 showed no clear trend in MVPA levels over time postpartum within this group.

#### 3.4.2 Group With MVPA Estimates ≥ 55.5 Min/Day

This group included five MVPA estimates that ranged from 55.5 to 87.3 min/day, measured from 0.46 to 8 months postpartum. Three of the estimates coming from two different studies [34, 37] used wrist‐worn ActiGraph devices that defined MVPA ≥ 100.6 mg; these two studies used the same cohort but reported MVPA from different periods postpartum. The two other estimates came from two studies [23, 35] that both defined MVPA ≥ 3 METs; however, they used different types and placement of PA trackers. In this group, there is a tendency of MVPA increasing gradually from 0.46 months until 8 months postpartum, with a total increase of 31.8 min/day over this period.

## 4 Discussion

The present systematic literature review summarizes findings of device‐measured PA throughout postpartum and found that estimates varied across different timespans in the postpartum period and between studies, along with differences in PA tracker types and placement, MVPA definitions, and participants characteristics. WHO recommends that the general adult population engage in at least 150 min MVPA per week to achieve various health benefits. This recommendation differs from the postpartum guidelines, of at least 150 min MPA per week, which can include VPA but does not require it [5]. This weekly recommendation corresponds to an average of approximately 21.4 min per day. While 21.4 min per day does not necessarily reflect a weekly amount of 150 min, due to daily variation, it offers a useful benchmark for interpreting the daily MVPA estimates reported in the included studies. Our analysis revealed substantial variation in MVPA estimates across studies, ranging from 2.14 to 87.3 min/day, with a predominantly bimodal distribution. To capture this pattern, MVPA estimates were categorized into three groups based on the observed ranges: ≤ 27.6 min/day, 27.6–55.5 min/day, and ≥ 55.5 min/day (Table 3). The highest and second lowest MVPA estimates in this review were measured in overlapping periods postpartum, with the highest estimate being more than 12 times greater than the second lowest. This substantial variability in estimates likely reflects a combination of factors, including differences in measurement methods, definitions of MVPA, and data management, as no gold standard currently exists for device‐measured PA [20, 46]. Additionally, variations in study populations, such as demographic characteristics, socioeconomic status, and differences in baseline PA levels further complicate comparison across studies.

Previous studies based on self‐reported PA have found that the transition to motherhood is associated with lower PA levels, both compared with pre‐pregnancy PA levels and compared with PA levels in nulliparous women. For instance, a systematic review from 2012 [10] reported that PA seems to decline from pre‐pregnancy to the postpartum period. Mottola et al. (2002) [47] proposed that PA levels rise between six and 12 months postpartum. Borodulin et al. (2009) [48] reported a decline in self‐reported PA during pregnancy with an increase 1 year after childbirth. Similarly, Pereira et al. (2007) [49] found that PA declined from pre‐pregnancy to pregnancy, followed by a partial rebound at 6 months postpartum. In addition, a recent population‐based study reported that Danish mothers aged 20–40 years had a 24% higher risk of non‐adherence to WHO PA guidelines compared with their nulliparous peers, suggesting that lower PA levels persist in the early years of motherhood [8]. However, limited research directly compares the findings of the present review. In this review, no clear trend in distribution was observed in the MVPA groups of ≤ 27.6 min/day and 27.6–55.5 min/day concerning time since childbirth (Figure 2). However, in the MVPA ≥ 55.5 min/day group (Figure 2), MVPA increased by 31.8 min/day from 0.46 to 8 months postpartum, suggesting a trend of increasing MVPA from early to later postpartum. These findings align with previous research indicating a potential increase in PA levels as women transition further into the postpartum period.

Based on the observed bimodal distribution, a clear pattern emerged regarding the definition of MVPA and PA tracker placement. In the group with MVPA ≤ 27.6 min/day, studies primarily used hip‐worn ActiGraph devices with the same or similar cpm thresholds. Only one study using a hip‐worn ActiGraph device measured higher MVPA than the rest, and that is Martin et al. (2017) [32], which was the only study in the intermediate MVPA group, with a weighted mean (SD) for MVPA at 44.7 (26.9) min/day. Typically, lower cpm thresholds for classifying MVPA result in longer reported duration of MVPA [25]. Perplexingly, Martin et al. (2017) [32] had the highest lower threshold among studies using cpm definitions. The relatively large combined standard deviation in Martin et al. (2017) [32] suggests considerable variability in MVPA within the study population, which could indicate that other factors, such as unique participant characteristics or differences in study design, could also contribute to this finding.

In contrast, there were no studies measuring MVPA using cpm in the MVPA ≥ 55.5 min/day group. Instead, two studies defined MVPA using METs, a definition only present in this group, and two studies using the definition MVPA ≥ 100.6 mg, with only one study in the MVPA ≤ 27.6 min/day group using this definition. This suggests that the measurement methods of MVPA may contribute to the observed heterogeneity in MVPA estimates. Furthermore, in the ≥ 55.5 min/day group, only one study placed the PA tracker at the hip, whereas most studies in the other groups had the tracker placed at the hip. While hip‐worn trackers are generally considered to provide more accurate measures [20], one included study reported similar activity counts for wrist‐ and hip‐worn accelerometers among postpartum women [36].

These methodological inconsistencies highlight the need for caution when interpreting findings from a single study, as the assessment of PA using wearable devices is complex and seems to depend on various factors. Previous papers have also emphasized the need for greater methodological alignment in PA research, to ensure greater homogeneity of outcome [17, 46, 50, 51].

Only two studies used consumer‐grade devices. Notably, these were the only studies that measured PA continuously for more than 1 week, and both reported average MVPA levels in the ≤ 27.6 min/day range. All other studies included used research‐grade devices, most of which measured PA over shorter periods (≤ 7 days). Although limited in number, the longer monitoring periods in the two consumer‐device studies suggest that such devices may have potential for being a feasible alternative to measure long‐term, real‐world PA assessment in postpartum populations.

Despite growing interest in postpartum PA, current literature predominantly has used self‐reported measures. A systematic review from 2021 on group‐based PA interventions for postpartum women highlighted the lack of data on device‐measured PA [50]. Similarly, several studies have emphasized this knowledge gap and the need for device‐based measures to better understand and promote health enhancing PA in this population [22, 31, 34, 52]. Most existing studies rely on self‐reported PA to describe the intensity of activities women do in the postpartum period. However, self‐reported PA is likely prone to recall bias [21, 44] and tends to overestimate PA levels compared to device‐based methods [19, 42].

The aim of the present review was to describe MVPA; however, studies that presented MPA and VPA separately were also included. This was done because the two estimates separately also provide meaningful information regarding the aim. It is worth noting that the mean duration of VPA reported was ≤ 1 min/day, suggesting that its exclusion from the figure likely had minimal impact on the overall presentation of estimates in Figure 2. While this approach is not entirely accurate, we deemed it the best approach given the available data.

The grouping of the MVPA estimates was done post hoc, based on observed ranges, to identify patterns in the otherwise heterogenetic data. The approach revealed a slight tendency for MVPA to increase from early to late postpartum periods, but only in the ≥ 55.5 min/day group. Additionally, although the grouping was based on observed ranges, the bimodal distribution may, in part, reflect differences in how PA was measured and defined across studies. Still, the grouping may oversimplify the complexity of MVPA patterns during the postpartum period.

The postpartum period begins at the birth of a newborn, but its duration lacks a clear definition [53]. In this review, a 1‐year timeframe was chosen, as it captures a broader range of physiological and behavioral changes postpartum [47, 53, 54, 55]. This longer period may provide a more comprehensive understanding of PA patterns following childbirth.

The focus of this review was on MVPA, in line with current WHO recommendations for postpartum women, which emphasize achieving at least 150 min of MPA per week. However, the WHO also states that doing some PA is better than doing none and recommends limiting sedentary behavior during the postpartum period to support health. Time spent being sedentary should be replaced with PA of any intensity, including light‐intensity physical activity (LPA) [5]. Previous literature has reported that postpartum women predominantly engage in LPA [8, 23, 34] with activities such as walking, strolling [8, 47, 56, 57] and caregiving [48]. As evidence grows supporting the health benefits of LPA [58, 59, 60], future research should explore whether new mothers derive meaningful benefits from the activities they are already engaging in and examine the role of LPA as a feasible starting point for increasing overall PA levels.

### 4.1 Strengths and Limitations

This systematic review has some limitations. First, only two databases (PubMed and Embase) were searched, which may have limited the scope of the review. However, this was supplemented by citation searching of the included studies' references and a Scopus search to identify additional studies. Second, only one author screened the studies, which may have introduced selection bias, despite predefined inclusion and exclusion criteria and the use of Covidence for structured screening. Third, measurement protocols varied across studies, with some assessing PA at specific postpartum time points and others using broader timeframes, complicating the comparability of estimates. Additionally, none of the studies defining MVPA by cpm used cut‐points specific to postpartum populations. Instead, cut‐points developed for adults were applied, which may not accurately reflect unique characteristics of postpartum women's thresholds, due to variations by weight, age, and sex [42].

However, the included studies instructed women to wear the PA trackers during waking hours, allowing measurement of all types of PA, including everyday tasks such as household work, work‐related PA, and childcare. This may be a strength of device‐measured PA compared to the self‐reported PA, which relies on the ability of questionnaires to capture all forms of PA [44]. But, PA trackers can be costly and depend on participants' compliance to wear the device consistently [17]. Additionally, included studies did not measure water‐based activities, potentially underestimating MVPA. However, it has been reported that water‐based activities decline from pre‐pregnancy and during pregnancy to the postpartum period [56, 57]. Third, device‐measured PA represents fair new technological advancements; therefore, studies published before 2000 were excluded to ensure that measured PA reflected current measurement technologies [17, 51]. This limits the generalizability of the findings to earlier periods. Fourth, generalizability was further limited due to the heterogeneity of sample demographics (e.g., specific BMI ranges, low‐income groups, or certain races) and small sample sizes, which further complicates cross‐study comparisons.

No formal quality assessment was conducted, due to the descriptive aim, inclusion of heterogeneous study designs, and the lack of validated tools to assess the quality of device‐measured PA in postpartum populations. This should be considered when interpreting the findings.

Initially, this present review aimed to describe MVPA postpartum in min/day or min/week. However, as only three studies [28, 30, 33] reported MVPA in min/week, the focus shifted toward MVPA in min/day. To enable comparison, MVPA estimates from these studies were converted to a min/day estimate by dividing min/week by seven. This method assumes an even distribution of PA throughout the week, which may not be entirely accurate, but it provides an idea of what the real mean duration of MVPA per day for these women may have been.

However, the comprehensive synthesis presented in this review offers novel and valuable insights into the insufficiently studied domain of postpartum PA, contributing to a deeper understanding of patterns, variations, and potential influencing factors in this population.

## 5 Perspectives

Future studies investigating device‐measured PA among postpartum women should carefully consider tracker type, placement, and cut‐points used to define PA, to enhance comparability. The largest sub‐group in this review used hip‐worn ActiGraph trackers and defined PA using cpm‐based cut‐points, mostly from Troiano et al. [42]. However, methodological differences in device type, wear location, and PA definitions across the included studies likely contributed to variability in MVPA estimates, thereby limiting comparability between studies. Standardizing PA measurement methods may improve comparability across studies and enhance our understanding of PA in the postpartum period and its role in maternal health.

Despite methodological challenges, this review provides a descriptive overview of the existing literature on device‐measured PA during the first year of motherhood. Notably, nine of the 15 included studies reported that postpartum women did not meet WHO recommendations for PA, suggesting that many new mothers may be insufficiently active and highlighting the need for interventions and further research to promote and understand maternal PA. Although this review focused on MVPA, previous studies suggest that many postpartum women primarily engage in LPA [8, 23, 34]. Future research should examine the association between LPA and health benefits and if it could serve as a starting point to increase overall activity levels.

By addressing current methodological inconsistencies and exploring the full range of postpartum PA, future studies can contribute to a more comprehensive understanding of how to promote sustained PA during early motherhood.

## Conflicts of Interest

The authors declare no conflicts of interest.

## Supporting information




Data S1–S3.

Data S4.

## Data Availability

The data that supports the findings of this study are available in the Supporting Information of this article.
